# Pemphigus Revealing Profound Hypothyroidism: A Rare Association

**DOI:** 10.7759/cureus.99565

**Published:** 2025-12-18

**Authors:** Oumaima Mandari, Youness El Khachine, Ali Halouache, Chayma A Hassani, Ikram Damoune, Abdelmajid Chraibi, Mohammed Tbouda, Lhoussaine Abainou

**Affiliations:** 1 Endocrinology, Diabetology and Metabolic Diseases, Mohammed VI University Hospital, Agadir, MAR; 2 Dermatology, Military Hospital Oued Eddahab, Agadir, MAR; 3 Endocrinology, Diabetology and Metabolic Diseases, Military Hospital Oued Eddahab, Agadir, MAR; 4 Pathology, Military Hospital Oued Eddahab, Agadir, MAR

**Keywords:** autoimmune bullous dermatosis, autoimmune hypothyroidism, pemphigus vulgaris, polyautoimmunity, hashimoto’s thyroiditis

## Abstract

Pemphigus vulgaris is a rare autoimmune bullous dermatosis, and while Hashimoto’s thyroiditis is the leading cause of hypothyroidism, their association falls within the spectrum of polyautoimmunity. A 50-year-old female patient with no specific past medical history presented with a four-month history of a clinical picture combining erosive mucocutaneous bullous lesions and significant asthenia. Further history-taking revealed that the patient exhibited significant psychomotor retardation, accompanied by subtle systemic signs (psychomotor slowness, bradycardia, and lower limb edema). A thyroid workup was performed accordingly, revealing severe hypothyroidism (ultra-sensitive thyroid-stimulating hormone > 68 IU/L, with severely depressed free T4 secondary to Hashimoto’s thyroiditis, which was confirmed by the positivity of anti-thyroid peroxidase antibodies and thyroid ultrasonography. The management initially combined general corticosteroid therapy with prednisone and hormone replacement therapy with levothyroxine, resulting in a favorable clinical outcome after two months of treatment for the pemphigus and the normalization of thyroid hormone levels. This association, although rare, is reported in the literature. It is explained by the existence of a common genetic background, notably involving certain HLA system haplotypes, as well as a global immune dysregulation favoring the emergence of multiple autoimmune pathologies in the same individual. This clinical observation highlights the importance of systematic screening for other autoimmune diseases, particularly thyroid disorders, in all patients with pemphigus.

## Introduction

Pemphigus vulgaris (PV) is a rare but potentially fatal autoimmune bullous dermatosis characterized by the formation of intraepidermal blisters due to autoantibodies directed against desmosomal proteins, leading to a loss of keratinocyte adhesion (acantholysis) [1].

Hashimoto’s thyroiditis (HT), conversely, is the most common autoimmune endocrine disease and a major cause of hypothyroidism worldwide. It results from the destruction of thyroid tissue by the immune system, primarily via humoral and cellular immune mechanisms. Although often asymptomatic in its early stages, HT can have significant systemic consequences and is frequently associated with other autoimmune diseases [2].

The coexistence of PV and HT is rare, yet clinically significant. This association raises important questions regarding shared autoimmune pathways, genetic predisposition, and underlying immune dysregulation. From a diagnostic standpoint, recognizing such associations may prompt clinicians to investigate for other autoimmune disorders when an index association is identified [3].

The objective of this case report is to describe a rare association between PV and HT in a middle-aged patient, to emphasize its clinical significance, and to explore potential underlying immunological mechanisms in light of current literature.

## Case presentation

**Figure 1:**
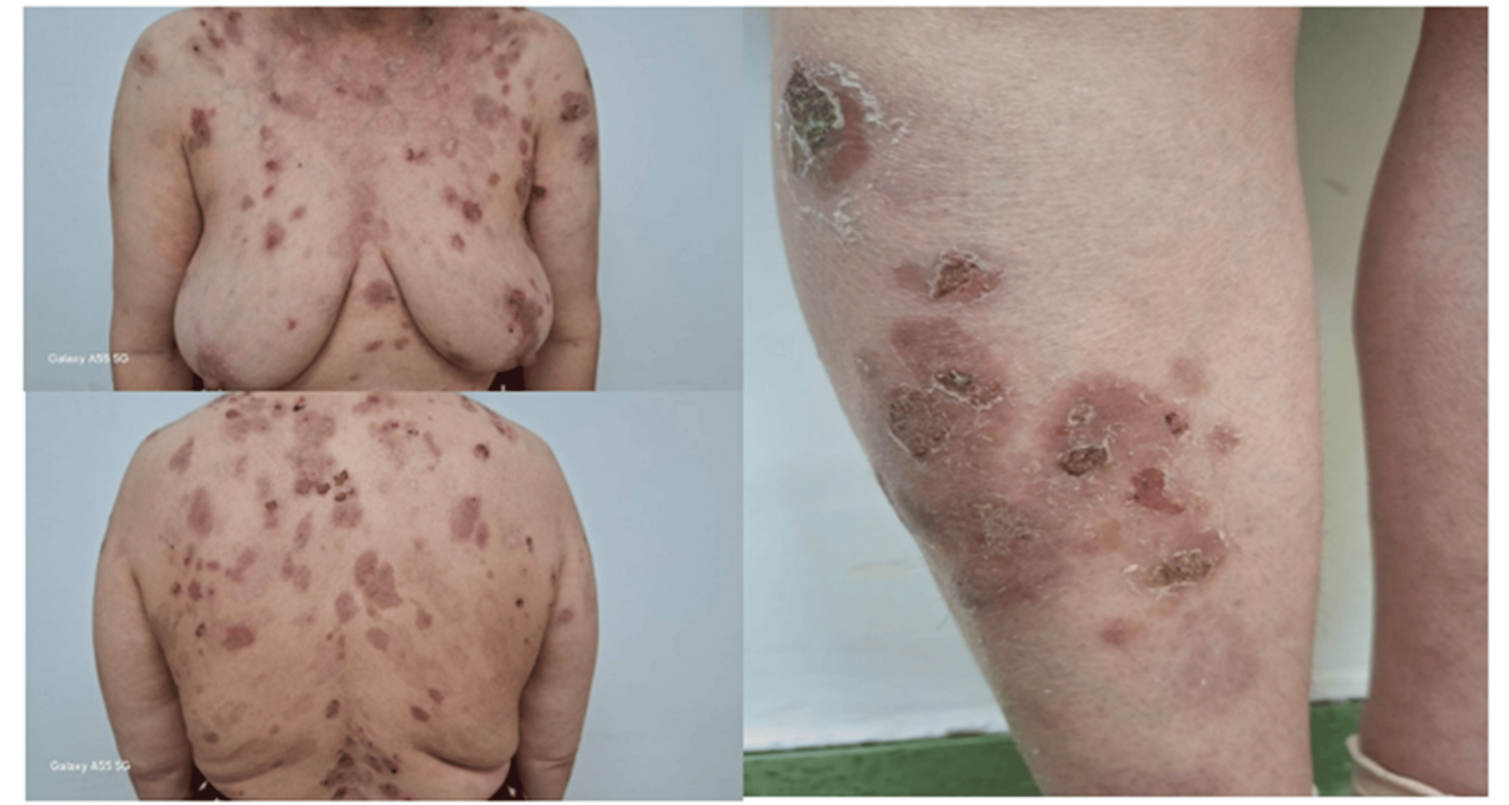
Erosive and crusted plaques on the trunk and leg.

A 50-year-old female patient with no specific past medical history presented with a four-month history of a clinical picture combining erosive mucocutaneous bullous lesions and significant asthenia. Dermatological examination revealed the presence of a bullous dermatosis consisting of flaccid bullae and post-bullous erosions, predominantly located on the trunk and limbs, with associated involvement of the oral and genital mucosa (Figure 1). There was no history of recent medication intake, recent infection, or atopic background.

**Figure 2:**
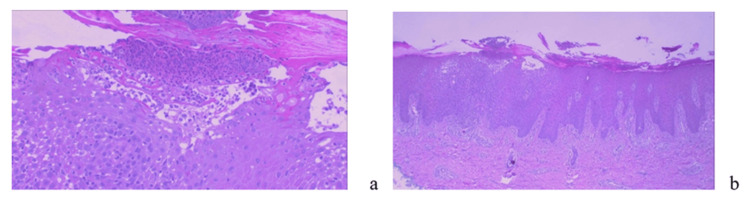
Skin biopsy results. (a) Skin covering showing an intraepidermal bulla (hematoxylin and eosin, ×100). (b) The bulla is intraepidermal, filled with neutrophils and eosinophils, with the presence of acantholytic cells (hematoxylin and eosin, ×200).

The patient was hemodynamically and respiratory stable but exhibited significant psychomotor retardation accompanied by subtle general signs: bradycardia at 60 beats/minute, blood pressure of 130/70 mmHg, a respiratory rate of 18 breaths/minute, and infiltrative edema of the lower limbs. The skin biopsy demonstrated an intraepidermal bullous dermatosis, suggestive of pemphigus (Figure 2).

The thyroid workup revealed profound hypothyroidism (ultra-sensitive thyroid-stimulating hormone (uTSH) at 68.49 µIU/mL, severely depressed free T4 at <0.4 ng/dL) of autoimmune origin, confirmed by a high positivity for anti-thyroid peroxidase (anti-TPO) antibodies (>1,000 IU/mL) and anti-thyroglobulin antibodies (85 IU/mL). Thyroid ultrasound revealed a heterogeneous thyroid gland, consistent with an autoimmune thyroiditis, which supported the diagnosis of HT. The metabolic workup revealed mixed dyslipidemia (hypercholesterolemia at 2.95 g/L and hypertriglyceridemia at 1.96 g/L). The inflammatory workup, tumor markers, and serum protein electrophoresis were all normal. The patient’s laboratory workup is summarized in Table 1.

**Table 1:** Laboratory results at admission.

Test	Result	Reference values
Ultra-sensitive thyroid-stimulating hormone	68.49 µIU/mL	0.4–4.0 µIU/mL
Free thyroxine	<0.4 ng/dL	0.8–1.8 ng/dL
Anti-thyroid peroxidase antibodies	>1,000 IU/mL	<35 IU/mL
Anti-thyroglobulin antibodies	85 IU/mL	<40 IU/mL
Total cholesterol	2.95 g/L	<2.00 g/L
Triglycerides	1.96 g/L	<1.50 g/L
Erythrocyte sedimentation rate	14 mm/h	<20 mm/h
C-reactive protein	0.9 mg/L	<5 mg/L
Carcinoembryonic antigen	3 µg/L	<5 µg/L
Cancer antigen 19-9	6.05 U/mL	<37 U/mL
Cancer antigen 15-3	30 U/mL	<30 U/mL
Cancer antigen 125	17 U/mL	<35 U/mL
Serum protein electrophoresis	Normal	Normal

The thoraco-abdomino-pelvic CT scan revealed no obvious tumor lesions, which ruled out a paraneoplastic etiology for the bullous dermatosis.

Systemic corticosteroid therapy with prednisone was initiated at a dose of 1 mg/kg/day (60 mg/day), combined with hormone replacement therapy using levothyroxine, which was started at 25 µg/day and then gradually adjusted up to 1.6 µg/kg/day. The outcome was favorable, with progressive healing of the skin lesions. After three months of follow-up, while on prednisone reduced to 20 mg/day and levothyroxine at 100 µg/day, a significant improvement in skin condition was observed, along with a marked regression of the clinical signs of hypothyroidism. The dermatologists then introduced an adjuvant immunosuppressive treatment with azathioprine (100 mg/day) to facilitate the gradual tapering of the corticosteroid therapy.

The patient’s clinical progress was favorable starting from the first month of follow-up, characterized by progressive healing of the skin lesions and a significant regression of the clinical signs of hypothyroidism. The patient was maintained on a reduced dose of prednisone (20 mg/day), combined with a hormone replacement therapy using levothyroxine at a dose of 100 µg/day. After three months of follow-up, a marked improvement in the cutaneous involvement was observed, accompanied by a normalization of the thyroid function tests, with the TSH measured at 3.56 mIU/L.

## Discussion

HT and pemphigus share a common origin rooted in a breakdown of immune tolerance [4]. Pemphigus is an autoimmune bullous dermatosis linked to anti-desmoglein autoantibodies. Several studies suggest an association with thyroid autoimmunity, specifically the presence of positive antithyroid autoantibodies and, in some cases, HT [4,5]. Our clinical observation perfectly illustrates this association.

A study conducted by Zeng et al. in 2022 included six studies involving a total of 17,567 patients with pemphigus. The results revealed a significant association between pemphigus and hypothyroidism, with a relative risk of 1.70 and a 95% confidence interval ranging from 1.54 to 1.87 [6].

Both autoimmune diseases involve the abnormal activation of T and B lymphocytes, which leads to the production of specific autoantibodies: anti-thyroperoxidase and anti-thyroglobulin in HT and anti-desmogleins in pemphigus [7]. In HT, this autoimmune response results in the progressive destruction of the thyroid parenchyma and hypothyroidism [8,9]. In pemphigus, it causes intraepidermal acantholysis, which is responsible for the formation of bullae and mucocutaneous erosions [1].

T lymphocytes, which are key regulators of adaptive immunity, play a central role in this process. Hypothyroidism, particularly when autoimmune (as seen in Hashimoto’s disease), induces a significant immune dysregulation. This condition disrupts the delicate balance among T lymphocyte subpopulations: it often promotes the pro-inflammatory activity of Th1 and Th17 lymphocytes, while simultaneously diminishing the regulatory action of Treg lymphocytes. This systemic immune imbalance, when combined with the direct effects of hormone deficiency on the skin, which include slowed skin metabolism, xerosis, and disruption of the protective barrier, creates a favorable environment for cutaneous eruption. The resulting compromised skin becomes more permeable to irritants or auto-antigens, triggering an inappropriate and localized inflammatory response mediated by these dysregulated T lymphocytes, which clinically manifests as a skin rash.

The association of these two pathologies is a form of polyautoimmunity, which reflects the existence of a common immunogenetic background. This background involves certain HLA system haplotypes (such as HLA-DR4) and immune regulation genes [5,6]. Screening for thyroid comorbidities in patients with pemphigus is essential, especially as about 25% of them are anti-TPO seropositive despite an initially normal TSH [6].

Therefore, according to international endocrinology guidelines, it is advisable to systematically measure TSH and anti-TPO antibodies, even in the absence of suggestive symptoms [10]. This approach allows for the early detection of autoimmune thyroiditis and the identification of patients at risk for thyroid dysfunction related to immunosuppressants [6]. It also enables therapeutic adjustments to be anticipated, especially when initiating high-dose corticosteroid therapy, which can mask or accentuate a pre-existing thyroid imbalance [5].

The management of this specific autoimmune association necessitates a rigorously coordinated multidisciplinary approach between a dermatologist and an endocrinologist. The first-line treatment for pemphigus is typically based on high-dose systemic corticosteroid therapy, often initiated at 1 mg/kg/day. This treatment may be combined with conventional immunosuppressants, such as azathioprine (100 mg/day), or with biotherapies in severe or resistant cases [1]. HT is treated primarily with thyroid hormone replacement therapy, using levothyroxine. The initial dosing for overt hypothyroidism is typically calculated at approximately 1.6 µg/kg/day [9]. For subclinical hypothyroidism, treatment is individualized, depending on factors such as TSH, anti-TPO levels, symptoms, pregnancy, goiter, and cardiovascular risk [11].

## Conclusions

The association between HT and PV highlights the concept of polyautoimmunity. Thus, a diagnosis of pemphigus should warrant screening for thyroid function, as well as for anti-TPO antibodies. It also underscores the importance of a systemic approach in patients with autoimmune diseases: the presence of one autoimmune condition must raise suspicion for other manifestations, even in distant and functionally different organs.
